# Brain reserve in memory regions is associated with the preservation of autobiographical memories after electroconvulsive therapy

**DOI:** 10.3389/fpsyt.2025.1699102

**Published:** 2025-11-10

**Authors:** André Beyer Mathiassen, Christoffer Cramer Lundsgaard, Krzysztof Gbyl, Kamilla Miskowiak, Birgitte Fagerlund, Henrik B. Wiberg Larsson, Ulrich Lindberg, Poul Videbech

**Affiliations:** 1 Center for Neuropsychiatric Depression Research, Mental Health Center Glostrup, Copenhagen University Hospital – Mental Health Services CPH, Glostrup, Denmark; 2 Department of Clinical Medicine, University of Copenhagen, Copenhagen, Denmark; 3 Neurocognition and Emotion Across Disorders of the Brain Centre (NEAD), Psychiatric Centre Copenhagen, Frederiksberg, Denmark; 4 Department of Psychology, University of Copenhagen, Copenhagen, Denmark; 5 Child and Adolescent Mental Health Center, Mental Health Services CPH, Copenhagen, Denmark; 6 Functional Imaging Unit, Department of Clinical Physiology and Nuclear Medicine, Copenhagen University Hospital – Rigshospitalet, Copenhagen, Denmark

**Keywords:** electroconvulsive therapy, amnesia, prediction, brain reserve, hippocampal volume, cortical thickness

## Abstract

**Background:**

Electroconvulsive therapy (ECT) may cause anterograde and autobiographical amnesia. Identifying reliable predictive biomarkers for the risk of this memory loss may assist clinicians in their decision to use this treatment. We aimed to investigate whether the baseline volume or the cortical thickness of the predetermined regions of interest (ROIs) involved in memory recall is correlated with the degree of amnesia after ECT.

**Method:**

In this longitudinal study, we followed 19 inpatients receiving ECT for depression. Using 3-T MRI, we performed structural brain scans prior to ECT. We also assessed the autobiographical, verbal, and visual anterograde memory both before and after ECT. We conducted one-sided partial correlation analyses between the volume or the cortical thickness of the ROIs at baseline and the memory decline following ECT (ClinicalTrials.gov: NCT04160286).

**Results:**

As hypothesized, a larger baseline cortical thickness of the right parahippocampal gyrus (*r* = 0.517, *p* = 0.014) and the left ventrolateral prefrontal cortex (*r* = 0.530, *p* = 0.012) correlated with less ECT-related decline in autobiographical memory, respectively explaining 26.7% and 28.1% of the variance. Unexpectedly, a smaller volume of the right hippocampus also correlated with less autobiographical memory decline (*r* = −0.416, *p* = 0.048). None of the ROIs predicted anterograde memory impairment.

**Conclusion:**

These early findings suggest that a pre-ECT neural reserve in the brain regions subserving memory might act protectively against the development of autobiographical memory loss after ECT. If replicated in larger samples, our findings may have promising clinical implications as a structural MRI scan prior to ECT might contribute to determining the individual risk of autobiographical memory loss following ECT.

## 1 Introduction

Electroconvulsive therapy (ECT) is remarkably effective in the treatment of severe uni- and bipolar depression (1) and is widely used across the globe (2, 3). Unfortunately, ECT is associated with anterograde and retrograde (autobiographical) amnesia, which may be severe in some cases (4–6). Therefore, the ability to predict such side effects prior to treatment initiation would be highly valuable in the clinical setting as it would enable clinicians to identify patients at high risk of developing adverse amnesic effects following ECT. This may facilitate the shared decision-making between patients, their relatives, and their physician.

The concept of *brain reserve* refers to the observation that larger brains tolerate more detrimental effects before cognitive impairment emerges (7, 8). Brain reserve has been found to be protective against cognitive impairment in relation to various pathological brain changes, including traumatic brain injury (8), multiple sclerosis (9), and Alzheimer’s disease (7). Brain volume and cortical thickness are considered direct measures of such brain reserve (10). Retrograde memory recall has been found to be positively correlated with the cortical thickness of the brain regions important for autobiographical memory function (11). These observations are further substantiated by functional imaging studies that found the activation of these and other pertinent regions to be correlated with autobiographical memory recall. Specifically, the recall of autobiographical memories has most frequently been associated with the hippocampus (HC), the medial prefrontal cortex (mPFC), the parahippocampal gyrus (PHG), the posterior cingulate cortex (PCC), the ventrolateral prefrontal cortex (vlPFC), and the angular gyrus (AG) (12–14). The anterograde memory function is positively correlated with the hippocampal volume in various patient populations, including depression (15, 16). Thus, it is plausible that a brain reserve in these regions may act protectively against the development of amnesia following ECT. If so, such a measure may have potential to serve as a useful predictive biomarker for this amnesia. To the best of our knowledge, this has never been investigated before.

### 1.1 Aims

The aim of this study was to explore potential predictive biomarkers in the brain for the risk of developing amnesia following ECT by studying how the baseline brain structure relates to this amnesia. Based on the literature, we hypothesized the following:

1. Less autobiographical amnesia will be correlated with a larger baseline hippocampal volume and a larger baseline thickness of the following cortical regions: mPFC, PHG, PCC, vlPFC, and AG (12–14).
2. Less anterograde amnesia will be correlated with a larger hippocampal volume at baseline. Verbal and visual anterograde amnesia will be more strongly correlated with the left and the right HC, respectively (17, 18).

## 2 Materials and methods

This manuscript was conducted in agreement with the Strengthening the Reporting of Observational Studies in Epidemiology (STROBE) guidance. The study was part of The Danish Neuropsychological Study of the Adverse Effects of ECT (DANSECT), which was registered on ClinicalTrials.gov (ID: NCT04160286) before patient recruitment commenced. It was approved by the Ethical Committee of the Capital Region of Denmark (H-19038861).

### 2.1 Study design and sample

DANSECT was designed as an extension of the Danish ECT/MRI project (ClinicalTrials.gov ID: NCT03040388), as the magnetic resonance imaging (MRI) techniques were the same as those in this project and complemented with neuropsychological measurements (19–23).

This longitudinal study is prospective and observational. We examined inpatients with moderate-to-severe depression who received a series of ECT. Patients were consecutively recruited from mental health centers in the Capital Region of Denmark between December 2020 and July 2023.

Inpatients between 18 and 95 years of age, fluent in Danish, and referred for ECT with moderate-to-severe uni- or bipolar depression according to ICD-10 (24) were eligible for inclusion. Patients with one or more of the following characteristics were excluded: 1) showing severe psychotic symptoms or suicidality, making transportation to MRI hazardous; 2) undergoing maintenance ECT or having already received ECT during the past 6 months; 3) with schizophrenia or other psychotic disorder, apart from psychotic depression; 4) with dependency syndrome cf. ICD-10 (24); 5) with a history of head trauma causing unconsciousness for more than 5 min; 6) with severe somatic or neurological illness affecting the brain; and 7) requiring compulsory treatment. Written and verbal informed consent was obtained from the patients prior to inclusion in our study.

The depression diagnosis giving rise to referral to this project was initially made by a psychiatrist at the psychiatric ward. Subsequently, this diagnosis was confirmed by the project neuropsychologist (AM) or the project medical doctor (CL) using the Mini-International Neuropsychiatric Interview (M.I.N.I.) (25). In order to verify that the symptoms of depression were not due to organic causes, a physical examination, blood tests, electrocardiogram, and urine screening for drugs were performed.

The MRI scans were conducted at pre-ECT (baseline): 1–2 days before ECT. The Wechsler Adult Intelligence Scale IV (WAIS-IV) Vocabulary (26) was applied at post-ECT: 5 (±2) days after the last ECT session. The remaining clinical assessments were performed at both pre- and post-ECT.

### 2.2 Clinical assessments

The depression severity was measured using the six-item Hamilton Rating Scale for Depression (HAM-D6) (27). The six-item version was chosen as it has been proven to be as sensitive as the longer versions while also being superiorly equidistant (28). Autobiographical memory was measured using the Columbia Autobiographical Memory Interview—Short Form (CAMI-SF) (29), which is the most frequently used tool for assessment of autobiographical memory performance in the ECT literature. Verbal anterograde memory was measured with the Verbal Learning Test—Delayed (VLT-D), which is a subtest of the Screen for Cognitive Impairment in Psychiatry (SCIP-D) (30). Parallel versions were employed at different time points. Visual anterograde memory was measured using the Rey Complex Figure Test (RCFT) 3-min recall (31). The RCFT was administered at both time points due to the different psychometric properties of alternative complex figures (32–34). Premorbid level of intelligence was assessed using the WAIS-IV Vocabulary.

### 2.3 MRI data

#### 2.3.1 MRI scanner

All MRI scans were carried out on a Philips Achieva dStream (Philips Healthcare, Best, Netherlands) with 3 T. The scanner was equipped with a phased-array receive-only head coil with 32 channels. High-resolution anatomical scans, both T1- and T2-weighted, were conducted using the following parameters: field of view = 256 × 240 × 180 mm^3^, acquisition time = 335 s, repetition time = 11 ms, echo time = 5.08 ms, inversion delay = 950 ms, flip angle = 8°, no. of slices = 257, turbo factor = 159, and voxel size = 0.7 × 0.7 × 0.7 mm^3^ for T1-weighted and field of view = 256 × 240 × 180 mm^3^, acquisition time = 305 s, repetition time = 2,500 ms, echo time = 330 ms, flip angle = 90°, no. of slices = 257, turbo factor = 113, and voxel size = 0.7 × 0.7 × 0.7 mm^3^ for T2-weighted.

#### 2.3.2 MRI data processing

MRI data were based on T1- and T2-weighted images. These were automatically processed in FreeSurfer (version 7.4.1) (35) using the longitudinal stream (36). Cortical parcellation was automatically performed using the Desikan–Kiliany (37) and the Destrieux atlas (38). The latter atlas was only used for parcellation of the AG as the Desikan–Kiliany atlas does not offer a parcellation corresponding appropriately to this region. Hippocampal segmentation was automatically conducted using the hippocampal module (39). Since the thickness of the perirhinal cortex is not automatically extracted in FreeSurfer, these values were manually extracted from *ex vivo* data. Quality control was performed through visual inspection of the coronal slices, ensuring correct delineation of the boundaries between gray and white matter. None of the slices gave rise to manual corrections.

#### 2.3.3 Regions of interest

##### 2.3.3.1 ROIs associated with autobiographical memory performance

**Figure 1:**
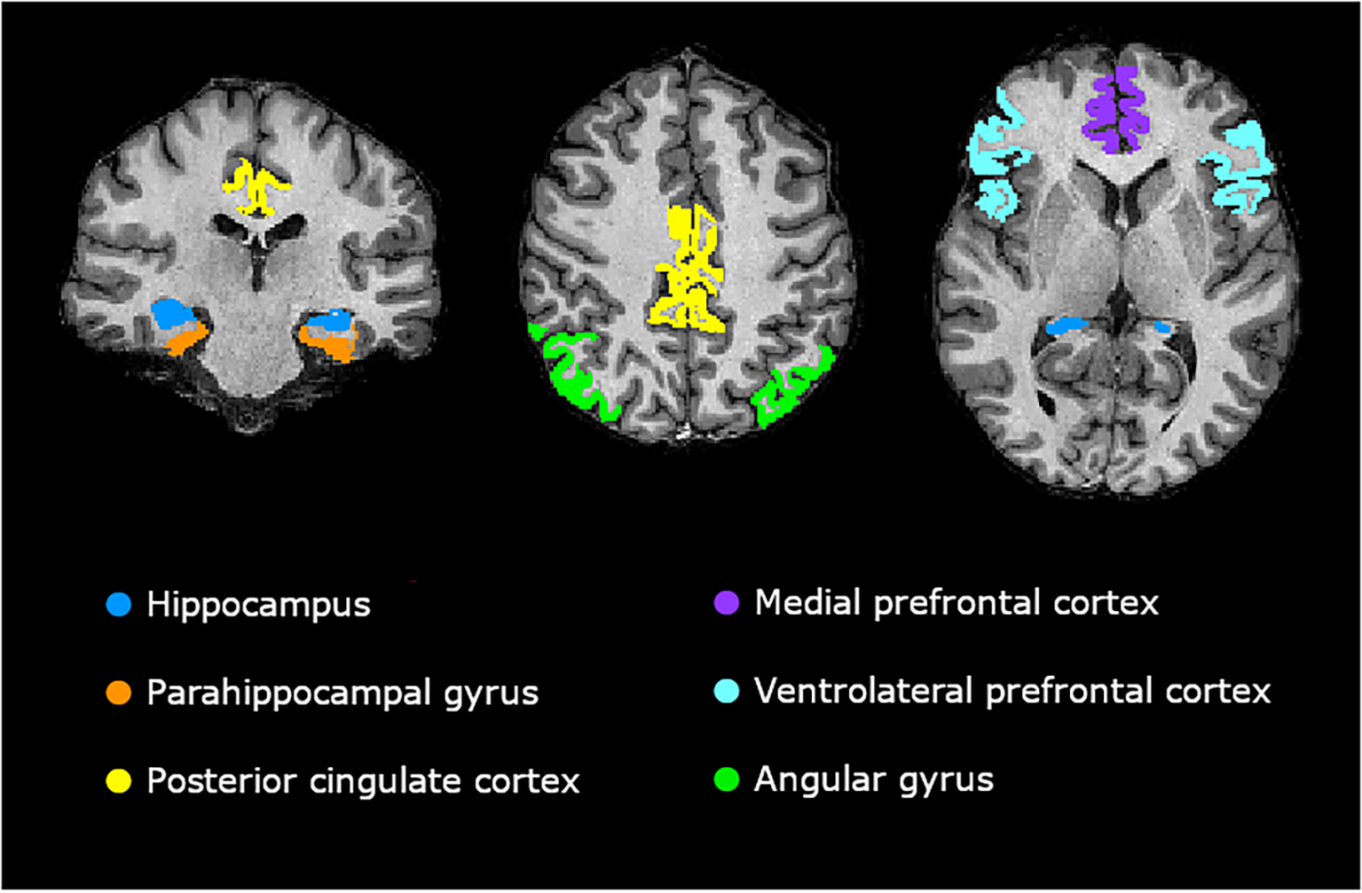
Visualization of the regions of interest (ROIs). Visualizations were rendered in FreeView (35).

The selection of the gray matter brain regions for which we expected the baseline volume or thickness to be associated with post-ECT changes in autobiographical memory performance was based on the convergence between three meta-analyses of functional MRI (fMRI) and PET studies (12–14). These studies attempted to identify the core brain regions involved in autobiographical memory recall. The selected regions of interest (ROIs) were the HC, PHG, PCC, mPFC, vlPFC, and AG. The PHG, mPFC, and vlPFC were composite measures comprising the following FreeSurfer parcellations: parahippocampal, entorhinal, and perirhinal cortices (PHG); medial orbitofrontal cortex and rostral anterior cingulate cortex (mPFC); and pars opercularis, triangularis, and orbitalis (vlPFC). **Figure 1** presents an overview of the selected ROIs.

##### 2.3.3.2 ROIs associated with anterograde memory performance

The selection of the HC as the ROI, for which we expected the baseline volume to be associated with the changes in anterograde memory performance after ECT, was based on the vast literature linking this structure to anterograde memory from the first study of patient H.M (17) until recent years (18).

### 2.4 Electroconvulsive therapy

The ECT treatment followed the standard clinical procedures of the Mental Health Service of the Capital Region of Denmark. Bilateral (bitemporal) electrode placement was applied as standard. However, the treating physician could change to right unilateral electrode placement if cognitive side effects were intolerable. Treatment was given three times per week. The treating physicians in the clinic made all decisions regarding patient treatment, and the research group did not participate in these.

ECT was delivered using a Thymatron IV apparatus. The initial treatment charge was calculated according to the half-age method, and the charges used in subsequent treatments were decided according to the clinical effect and the seizure quality as evaluated using electroencephalograms. The pulse width used was brief pulse (0.5–1.0 ms). Anesthesia was performed with thiopental (2–4 mg/kg) and muscle relaxation with suxamethonium (0.75 mg/kg).

### 2.5 Statistical analyses

As all hypotheses had specific *a priori* directional predictions based on prior literature, we reported one-sided *p*-values. Both our hypotheses were tested with partial correlation analyses, conducted using SPSS (version 29.0.1.0) (40). All analyses were controlled for age due to an expectation of age-related atrophy. The analyses involving hippocampal volume were controlled for baseline total brain volume, while the analyses involving cortical thickness were not as cortical thickness is largely independent of head size (https://surfer.nmr.mgh.harvard.edu/fswiki/eTIV). The variables representing amnesia following ECT were computed as the percentage change from pre- to post-ECT (Δ%) as follows:


$$\Delta \%=\;\left(Mem_{post}-\;Mem_{pre}\right)/Mem_{pre}*\;100$$

where Mem_post_ is the post-ECT memory score and Mem_pre_ is the pre-ECT memory score.

We correlated the baseline volume or thickness of the ROIs with Δ% in autobiographical memory performance, Δ% in verbal, and Δ% in visual anterograde memory performance. For explorative purposes, these analyses were also run with the subregions (individual parcels) constituting the ROI in question. This was done in order to examine potential drivers of the association between ROI and amnesia.

For an overview of the applied independent and dependent variables, see **Supplementary Table S1**.

## 3 Results

### 3.1 Patient inclusion

**Figure 2:**
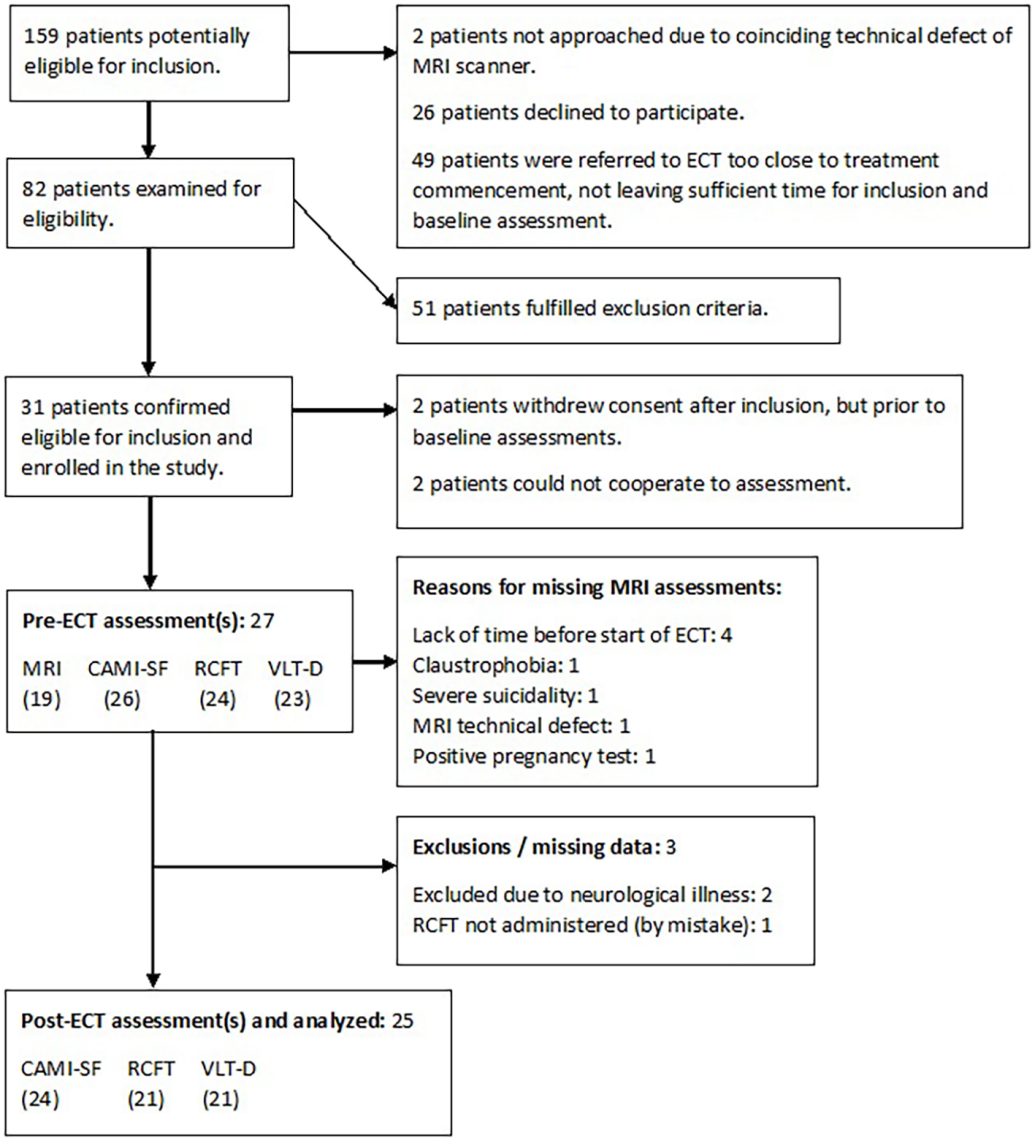
Flowchart of the patient inclusion process. *CAMI-SF*, Columbia Autobiographical Memory Interview—Short Form; *RCFT*, Rey Complex Figure Test; *VLT-D*, Verbal Learning Test—Delayed.

For an overview of the inclusion process, see the flowchart in **Figure 2**.

### 3.2 Demographic and clinical characteristics

The demographic and clinical characteristics of the included subjects are listed in **Table 1**.

**Table 1:** Demographic and clinical characteristics of the included subjects.

Demographics	
No. of subjects^a^	19
Age (years), mean (SD)	42.4 (13.4)
Gender, male/female	10/9
Estimated IQ^b^, mean (SD)	97.1 (11.9)
Handedness, left/mixed/right	1/1/17
Psychometry at baseline, mean (SD)	
HAM-D6 score	14.3 (2.3)
HAM-D17 score	26.6 (5.4)
CAMI-SF score	52.2 (4.3)
VLT-D score	6.8 (2.2)
RCFT score	20.1 (6.4)
ECT parameters	
No. of patients treated with BL ECT^c^	19
No. of patients treated with RUL ECT^c^	1
No. of ECT sessions, mean (SD)	11.1 (3.0)
Total seizure time (s), mean (SD)	469.3 (184.3)
Total dose (mC), mean (SD)	2,800 (2,656)
Antidepressant effect of ECT, *n* (%)	
No. of responders^d^	13 (68.4)
No. of remitters^e^	6 (31.6)

*ECT*, electroconvulsive therapy; *SD*, standard deviation; *HAM-D6*, six-item Hamilton Rating Scale for Depression; *CAMI-SF*, Columbia Autobiographical Memory Interview—Short Form; *VLT-D*, Verbal Learning Test—Delayed; *RCFT*, Rey Complex Figure Test; *BL*, bilateral electrode placement; *RUL*, right unilateral electrode placement.

a Subjects who completed the MRI scan.

b Estimated IQ was based on the Wechsler Adult Intelligence Scale (WAIS) Vocabulary score.

c All patients received ECT with bilateral electrode placement, and one patient also partially received right unilateral ECT.

d Response was defined as a reduction of ≥50% or more on the HAM-D6 score after ECT.

e Remission was defined as a HAM-D6 score ≤4 after ECT.

### 3.3 Memory decline following ECT

**Figure 3:**
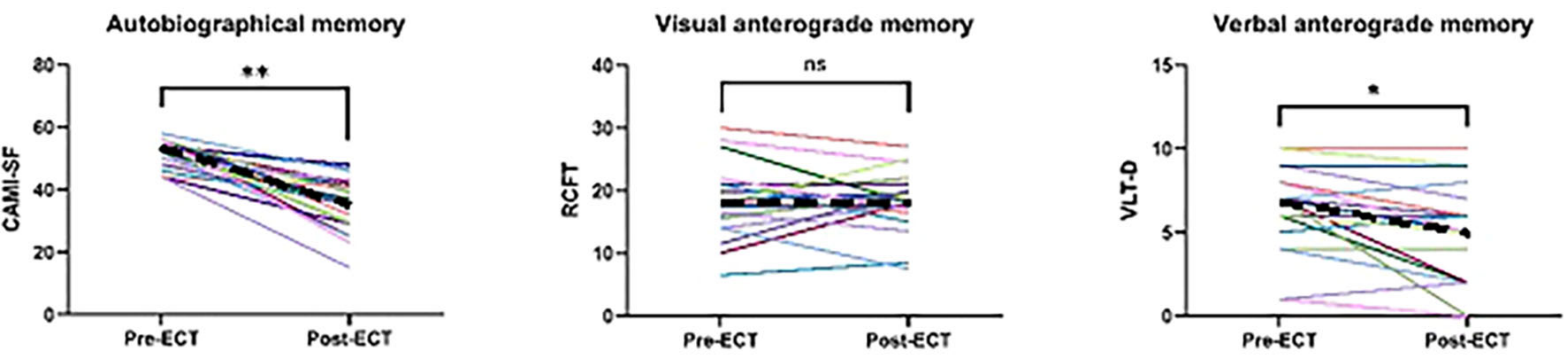
Change in memory performance from pre- to post-electroconvulsive therapy (ECT) across memory modalities. *CAMI-SF*, Columbia Autobiographical Memory Interview—Short Form; *ns*, not significant; *RCFT*, Rey Complex Figure Test; *VLT-D*, Verbal Learning Test—Delayed. *p = 0.003; **p < 0.001. *Dashes* denote the median value.

Performance on all three memory tests was approximately normally distributed based on inspection of the histograms. Paired-samples *t*-tests showed that both autobiographical [*t*(23) = 9.146, one-sided *p* < 0.001] and verbal anterograde [*t*(20) = 3.101, one-sided *p* = 0.003) memory performance decreased significantly from pre- to post-ECT on group levels. In contrast, there was no significant difference between pre- and post-ECT visual anterograde memory performance on group level (one-sided *p* = 0.352). For an overview of the individual trajectories, see **Figure 3**. Post-ECT memory assessment was conducted 177 h after the last ECT session, on average (SD = 141, range = 51–601).

### 3.4 Prediction of memory decline from baseline brain volume and cortical thickness

For an overview of the main results, see **Table 2**.

**Table 2:** Correlation analyses between baseline regions of interest (ROIs) and post-electroconvulsive therapy (ECT) memory loss.

	ROI and memory type	LH		RH	
		*r*	*p*	*r*	*p*
Hypothesis 1	Baseline volume or thickness and autobiographical memory loss				
	Hippocampus	−0.273	*0.145*	−0.416	***0.048***
	Parahippocampal gyrus	0.212	*0.199*	0.517	***0.014***
	Entorhinal cortex	0.009	*0.489*	0.565	***0.007***
	Perirhinal cortex	0.303	*0.111*	0.483	***0.021***
	Parahippocampal cortex	0.264	*0.145*	0.209	*0.202*
	Posterior cingulate cortex	−0.004	*0.493*	0.269	*0.141*
	Medial prefrontal cortex	0.142	*0.287*	0.368	*0.066*
	Medial orbitofrontal cortex	0.091	*0.359*	0.302	*0.111*
	Rostral anterior cingulate cortex	0.126	*0.309*	0.254	*0.154*
	Ventrolateral prefrontal cortex	0.530	***0.012***	0.247	*0.162*
	Pars opercularis	0.266	*0.143*	0.258	*0.150*
	Pars triangularis	0.177	*0.241*	0.311	*0.104*
	Pars orbitalis	0.681	***<0.001***	0.078	*0.379*
	Angular gyrus	0.019	*0.470*	0.374	*0.063*
Hypothesis 2	Baseline volume of the hippocampus and anterograde memory impairment				
	Verbal	0.039	*0.441*	0.077	*0.385*
	Visual	0.159	*0.278*	0.151	*0.289*

All *p*-values are one-sided.

*LH*, left hemisphere; *RH*, right hemisphere. Significant p-values (< 0.05) are bolded.

#### 3.4.1 Prediction of autobiographical memory loss

**Figure 4:**
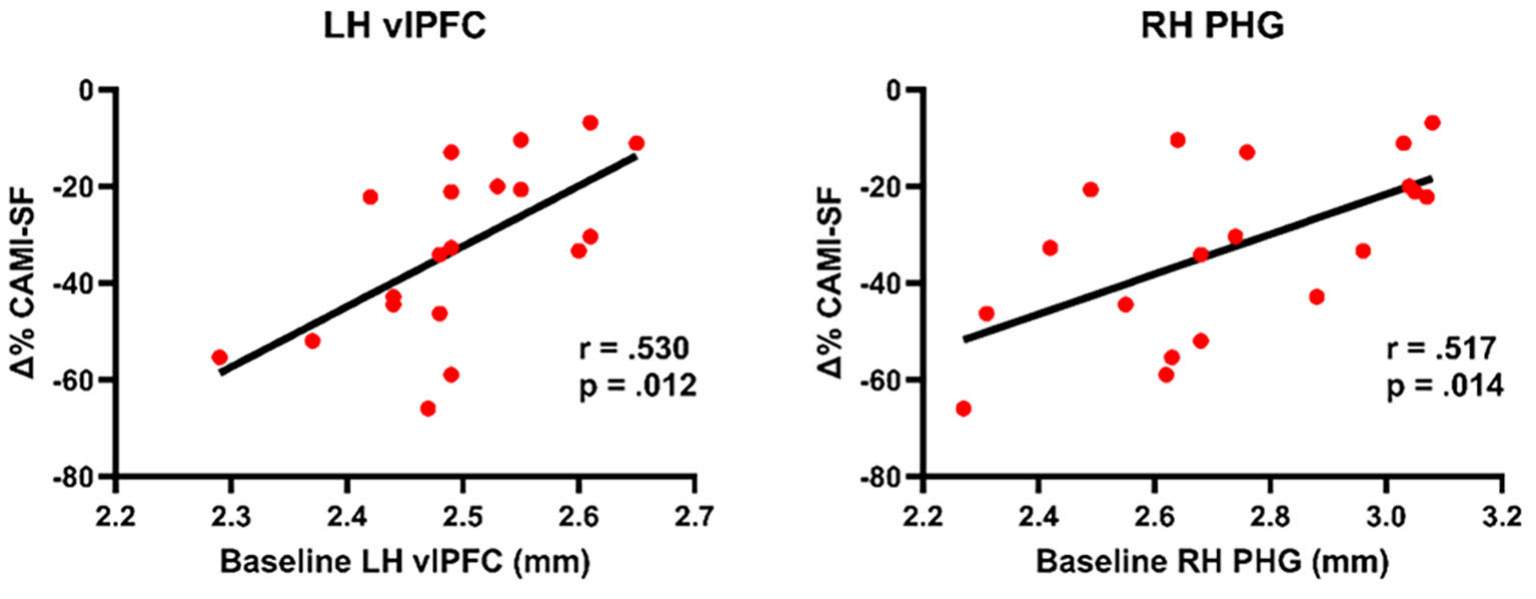
Baseline cortical thickness and post-electroconvulsive therapy (ECT) autobiographical memory loss. *CAMI-SF*, Columbia Autobiographical Memory Interview—Short Form; *LH*, left hemisphere; *PHG*, parahippocampal gyrus; *RH*, right hemisphere; *vlPFC*, ventrolateral prefrontal cortex; *Δ%*, percentage change from pre- to post-ECT. *P*-values are one-sided. The scatterplots are not controlled for patient age.

The baseline hippocampal volume and the thickness of the cortical ROIs showed approximate normal distributions based on inspection of the histograms. Less severe post-ECT autobiographical memory loss was correlated with a larger baseline thickness of the left vlPFC (*r* = 0.530, *p* = 0.012, *R*^2^ = 0.281) and the right PHG (*r* = 0.517, *p* = 0.014, *R*^2^ = 0.267) (see **Figure 4**). The correlation with vlPFC was driven by its anterior subregion, pars orbitalis (*r* = 0.681, *p* < 0.001, *R*^2^ = 0.464), while the PHG was driven by the subregions entorhinal (*r* = 0.565, *p* = 0.007, *R*^2^ = 0.319) and perirhinal (*r* = 0.483, *p* = 0.021, *R*^2^ = 0.233) cortices. In the opposite direction of the analyses above, and against our hypothesis, less severe autobiographical memory loss was correlated with a smaller baseline hippocampal volume in the right hemisphere (*r* = −0.416, *p* = 0.048, *R*^2^ = 0.173).

#### 3.4.2 Prediction of anterograde memory impairment

In contrast to our second hypothesis, the baseline hippocampal volume did not correlate with the degree of anterograde memory loss in any combination between laterality and either visual or verbal anterograde memory (*p* > 0.28 in all cases).

### 3.5 Post-hoc analyses

To explore whether the antidepressant effect of ECT could mediate our results, partial correlation analyses were performed between the baseline volume or the cortical thickness of the individual ROIs and the post-ECT change in HAM-D6. All analyses were controlled for age, and additionally the baseline total brain volume in the case of HC. We found a trending correlation between a better antidepressant effect and a smaller baseline volume of both the left HC (*r* = 0.470, two-sided *p* = 0.06) and the right HC (*r* = 0.466, two-sided *p* = 0.06). No significant correlations were observed with the cortical ROIs, but a trend was found with the left PCC (*r* = −0.465, *p* = 0.052).

To investigate whether floor effects in autobiographical memory performance potentially influenced our results, we examined whether the baseline volume of the right HC (controlled for age and baseline total brain volume) and the cortical thickness of the right PHG and left vlPFC (controlled for age) were correlated with the baseline autobiographical memory performance. A larger baseline right hippocampal volume was found to be correlated with better autobiographical memory performance at baseline (*r* = 0.559, two-sided *p* = 0.02). The baseline thickness of the cortical ROIs did not correlate with baseline performance (two-sided *p* ≥ 0.65 in both cases).

To explore whether the baseline thickness of the rest of the cortex was associated with the degree of autobiographical memory loss, correlation analyses were extended to all remaining regions parcellated by the Desikan–Kiliany atlas. This yielded significant correlations with the following regions: right insula, lateral orbitofrontal cortex, fusiform gyrus, supramarginal gyrus, inferior temporal gyrus, banks of the inferior temporal sulcus, and bilateral middle temporal gyrus (see **Supplementary Figure S1**).

An independent samples *t*-test showed no gender differences in the baseline cortical thickness of the left vlPFC or the right PHG (two-sided *p* ≥ 0.65 in both cases).

## 4 Discussion

Overall, we found that structures in both hemispheres correlated with the degree of post-ECT memory loss. Specifically, we found, as hypothesized, that a larger baseline thickness of the right PHG and the left vlPFC correlated with less severe autobiographical memory loss. Unexpectedly, we also found that a lower baseline volume of the right HC correlated with less severe autobiographical memory loss. We did not find hippocampal volume to be associated with either visual or verbal anterograde memory impairment.

The ROIs that were correlated with autobiographical memory loss were located in both hemispheres, and we found additional trending correlations in the right hemisphere (mPFC and AG). Furthermore, the *post-hoc* analyses almost exclusively showed right hemisphere correlations. This is interesting as the supratentorial neural correlates of this memory modality are predominantly found to be left-lateralized in the literature (14, 41). The association between autobiographical memory loss and the right hemisphere in our data could reflect the nature of autobiographical amnesia after ECT, which typically presents as a reversed gradient, i.e., recently established memories are more vulnerable to loss after ECT (4). This predominantly episodic, rather than semantic, memory loss occurring after ECT could explain the importance of the right hemisphere, as this hemisphere appears to be more involved in episodic recall than the left hemisphere, according to lesion studies (42, 43). It could also be a contributing factor that our patients’ autobiographical recall experiences are more emotionally intense due to their depression. This could explain the strong signal from the right hemisphere, as the literature has shown that the right hemisphere is recruited in addition to the left when recalling emotionally intense autobiographical memories (14), and when the memories elicit negative emotions in particular, there is evidence for a right-hemisphere dominance (44).

### 4.1 Hypothesis 1: prediction of autobiographical memory decline after ECT

Overall, we found partial support of our first hypothesis as the degree of post-ECT autobiographical memory decline was correlated with the baseline cortical thickness of several of the predetermined ROIs.

We found that a larger baseline thickness of the right PHG, driven by entorhinal and perirhinal cortices, and the left vlPFC, driven by the pars orbitalis, appeared to protect against severe autobiographical memory loss. This might reflect that a relatively larger structure constitutes a neural reserve and, therefore, a relative resilience toward developing autobiographical amnesia after ECT, in accordance with the threshold theory (8). The relevance of these memory regions in relation to ECT-induced autobiographical memory loss is corroborated by *post-hoc* analyses showing that the thickness of only a small part of the cortex, including these regions, is related to the memory loss. The direction of the correlations is in accordance with the literature generally showing associations between a larger relative size of the cerebral regions involved in autobiographical memory and better autobiographical memory function (45–47). In contrast, we also found a smaller baseline volume of the right HC to be correlated with less severe autobiographical memory decline after ECT. Importantly, however, this result cannot formally serve as evidence against the null hypothesis as the direction of the correlation was opposite to what was hypothesized. Accordingly, the following considerations are merely exploratory. The unexpected direction could be explained by a floor effect, i.e., an inferior autobiographical memory recall at baseline could entail that there are fewer memories and details to lose over time, let alone to detect psychometrically. This explanation is supported by the *post-hoc* analyses showing that a smaller baseline right hippocampal volume, and not cortical thickness, was strongly correlated with inferior autobiographical memory performance at baseline. The antidepressant effect of ECT could potentially also mediate the finding as a smaller hippocampal volume has been found to predict a better antidepressant effect of ECT (19), possibly leading to a more favorable cognitive outcome. Accordingly, a *post-hoc* analysis of our data indicated a trend toward the antidepressant effect being larger in subjects with a smaller HC, albeit an actual mediation analysis was not performed due to insufficient statistical power. No significant correlations were observed between the antidepressant effect and the cortical ROIs, although a trend was observed with the left PCC. This might reflect that the antidepressant effect did not confound the correlation between regional cortical brain reserve and autobiographical memory loss.

Our results might reflect that the entorhinal and perirhinal cortices are important to the mechanism behind ECT-induced autobiographical amnesia. In accordance with this, the entorhinal and perirhinal cortices have earlier been linked to retrograde and subordinately autobiographical memory function (48, 49). The entorhinal cortex is regarded as a hub in the communication between the HC and the neocortex and has been found to be involved in long-term memory retrieval, supposedly extending beyond hippocampal involvement (49). Furthermore, animal studies have found perirhinal lesions in combination with entorhinal lesions to cause more extensive retrograde amnesia than entorhinal lesions alone (50). Therefore, it seems plausible that a pre-ECT neural reserve in these regions serves protectively against post-ECT amnesia.

The finding that a larger baseline thickness of the left vlPFC correlated with less autobiographical memory loss after ECT may suggest that the vlPFC plays an important role in the mechanism behind ECT-induced autobiographical amnesia. This is plausible as the vlPFC has been found to be essential to the active retrieval of autobiographical memories (51). Furthermore, the fMRI literature has shown that, when recalling autobiographical memories, the vlPFC is increasingly active the more recently established the memory is (52). Similarly, ECT-related autobiographical amnesia appears more pronounced for the most novel memories (temporal gradient) (4), thus substantiating the relevance of the vlPFC. The increased vlPFC activation with more recent autobiographical memory recall is in fact particularly the case with the anterior vlPFC (52), which corresponds to our finding that the pars orbitalis was the driver of the correlation.

### 4.2 Hypothesis 2: prediction of anterograde memory impairment after ECT

Our results did not support this hypothesis as the baseline hippocampal volume was not associated with the degree of anterograde memory decline after ECT. Our best attempt at explaining this result is the possibility that the hippocampal volume, in fact, might not reliably reflect anterograde memory ability in neurologically intact middle-aged people as the positive correlation from the literature emerges with age-related atrophy and neurological disease (15). Weak or even negative correlations have been observed in younger healthy adults (15). While a recent meta-analysis has provided partial rebuttal for this position, as it substantiates the positive correlation in healthy children and adolescents (53), the variance in anterograde memory performance explained by the hippocampal volume does seem to be considerably lower even in healthy elderly people compared with same-aged patients with mild cognitive impairment or Alzheimer’s dementia (54). Another possible explanation as to why we did not find a correlation between hippocampal volume and anterograde memory impairment could be the suboptimal statistical power associated with the relatively small sample size of this study.

## 5 Strengths and limitations

The relatively small sample size was the main limitation. Nevertheless, as our findings are coherent with established knowledge about the human memory function and its neuroanatomical foundation and as all of our findings are moderate or strong correlations, we expect to incite other researchers to attempt replicating our results in larger samples. It is also important to note that the results were not false discovery rate (FDR)-corrected since the purpose of the study was exploratory.

The observational design and the lack of a control group introduced a risk of a confounding effect due to varying simultaneous medications and potentially biased ratings. Nonetheless, the observational design does contain a high degree of ecological validity. With regard to the lack of a control group, the observed memory changes may not entirely be attributable to ECT. In the case of autobiographical memory, a degree of memory loss is expected due to the passage of time and to depression. In the case of the anterograde memory tests, improvements due to test–retest effects might have masked an association with hippocampal volume.

To assess autobiographical memory performance, we applied the most frequently used outcome measure in the field, the CAMI-SF. While its validity has been debated, we argue that the instrument has several advantages in the assessment of autobiographical amnesia in this population as the alternatives lack either ecological validity or sensitivity to recent episodic memory loss (6). However, we also argue that the sensitivity of the CAMI-SF could be improved (6) and recognize that the instrument has not undergone formal validation (55–58).

## 6 Conclusion

We found partial support for our first hypothesis, as a larger baseline cortical thickness of the right PHG and the left vlPFC correlated with less severe autobiographical memory decline after ECT. These early findings may reflect that a neural reserve in these specific brain areas serves as a protective mechanism against the development of autobiographical memory loss after ECT. This raises the question of whether these measurements have potential to be used as predictive biomarkers for the risk of severe autobiographical amnesia following ECT. However, the findings cannot be generalized at present as they would need to be replicated in larger samples.

The future implications of our findings, if replicated, appear promising as the application of a structural MRI scan before treatment commencement could potentially aid clinicians in identifying patients at high risk of developing autobiographical amnesia following treatment. Ultimately, this information would qualify the shared treatment decision-making and possibly inform the adjustment of treatment parameters to limit disproportional amnesic side effects. Finally, the observed neural correlates might enable future theories of the mechanism underlying amnesia following ECT.

## Data Availability

The raw data supporting the conclusions of this article will be made available by the authors, without undue reservation.
