# Supplementary material for: Brain reserve in memory regions is associated with the preservation of autobiographical memories after electroconvulsive therapy
Source: Front Psychiatry. 2025 Nov 10;16:1699102. doi: 10.3389/fpsyt.2025.1699102 (PMC12640906; doi:10.3389/fpsyt.2025.1699102)
Supplement: Supplementary file 1 [file DataSheet1.docx]

| **Table S1.** Overview of the variables applied in the statistical analyses | | |
| --- | --- | --- |
| **Hypothesis** | **Independent variables (ROIs)** | **Dependent memory variables** |
| 1 | Baseline UL ROIs and their subregions | ∆% autobiographical memory |
| 2 | Baseline UL hippocampus | ∆% visual anterograde memory  ∆% verbal anterograde memory |
| *Notes.* ROIs = Regions of interest; UL = unilateral; ∆% = percentage change. | | |

| **Figure S1.** Baseline cortical thickness and autobiographical memory loss |
| --- |
| 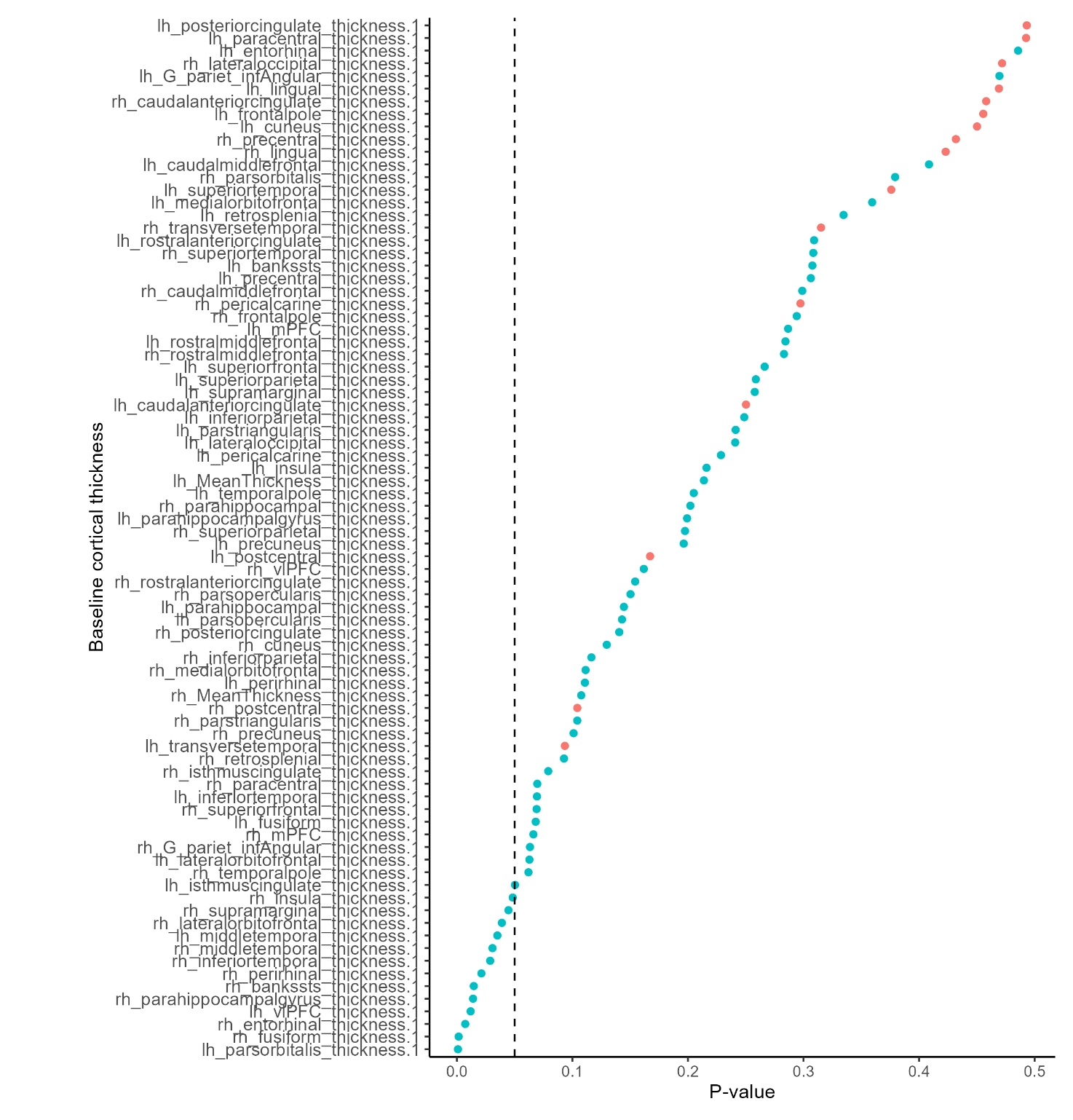 |
| *Notes.* This figure provides an overview of the one-tailed p-values of the correlations between post-ECT autobiographical amnesia and baseline thickness of each cortical region included in the Desikan-Kiliany atlas of FreeSurfer. All correlation analyses were partial and controlled for age. Blue-colored dots represent positive correlations, and red-colored dots represent negative correlations. The analyses were conducted in R version 4.4.1 [1–3]. |

**References**

[1] Wickham H, Averick M, Bryan J, Chang W, McGowan L, François R, et al. Welcome to the Tidyverse. J Open Source Softw 2019;4:1686. https://doi.org/10.21105/joss.01686.

[2] S K. _ppcor: Partial and Semi-Partial (Part) Correlation_ 2015.

[3] R Core Team. A language and environment for statistical computing n.d.
